# Overexpression of karyopherin-α2 in cholangiocarcinoma correlates with poor prognosis and gemcitabine sensitivity via nuclear translocation of DNA repair proteins

**DOI:** 10.18632/oncotarget.15020

**Published:** 2017-02-02

**Authors:** Mariko Tsukagoshi, Kenichiro Araki, Takehiko Yokobori, Bolag Altan, Hideki Suzuki, Norio Kubo, Akira Watanabe, Norihiro Ishii, Yasuo Hosouchi, Masahiko Nishiyama, Ken Shirabe, Hiroyuki Kuwano

**Affiliations:** ^1^ Department of General Surgical Science, Gunma University Graduate School of Medicine, Maebashi, Gunma 371-8511, Japan; ^2^ Department of Hepatobiliary and Pancreatic Surgery, Gunma University Graduate School of Medicine, Maebashi, Gunma 371-8511, Japan; ^3^ Division of Hepatobiliary and Pancreatic Surgery, Integrative Center of General Surgery, Gunma University Hospital, Maebashi, Gunma 371-8511, Japan; ^4^ Department of Molecular Pharmacology and Oncology, Gunma University Graduate School of Medicine, Maebashi, Gunma 371-8511, Japan; ^5^ Department of Surgery and Laparoscopic Surgery, Gunma Prefecture Saiseikai-Maebashi Hospital, Maebashi, Gunma 371-0821, Japan

**Keywords:** KPNA2, cholangiocarcinoma, gemcitabine, MRN complex, DNA repair

## Abstract

Cholangiocarcinoma is a highly malignant tumor, and the development of new therapeutic strategies is critical. Karyopherin-α2 (KPNA2) functions as an adaptor that mediates nucleocytoplasmic transport. Specifically, KPNA2 transports one of the important DNA repair machineries, the MRE11-RAD50-NBS1 (MRN) complex, to the nucleus. In this study, we clarified the significance of KPNA2 in cholangiocarcinoma. KPNA2 expression evaluated by immunohistochemical analysis was common in malignant tissue but rare in adjacent noncancerous tissues. KPNA2 overexpression was significantly correlated with poor prognosis and was an independent prognostic factor after surgery. In patients with cholangiocarcinoma who received gemcitabine after surgery, KPNA2 overexpression tended to be a prognostic indicator of poor overall survival. In KPNA2-depleted cholangiocarcinoma cells, proliferation was significantly decreased and gemcitabine sensitivity was enhanced *in vitro* and *in vivo*. Expression of KPNA2 and the MRN complex displayed colocalization in the nucleus. In addition, nuclear localization of the MRN complex was regulated by KPNA2 *in vitro*. These results suggest that KPNA2 expression may be a useful prognostic and predictive marker of gemcitabine sensitivity and survival. The regulation of KPNA2 expression may be a new therapeutic strategy for cholangiocarcinoma.

## INTRODUCTION

Cholangiocarcinoma is a malignant tumor originating from the bile duct epithelial cells that is classified as intrahepatic or extrahepatic depending on location [1, 2]. Although extrahepatic cholangiocarcinoma (EHCC) is relatively uncommon in Western countries, the incidence and mortality rate of cholangiocarcinoma continue to increase worldwide [3, 4]. In Japan in particular, it was reported that cholangiocarcinoma was responsible for approximately 12,000 deaths in 2013 [5]. Surgical resection is currently the only curative treatment for cholangiocarcinoma. However, the majority of patients are inoperable because most are diagnosed at an advanced stage [6–8]. Despite recent advances in surgical techniques and chemotherapy, the post-resection 5-year survival rate is only 20%–30% [9].

Gemcitabine (2′, 2′-difluoro-2′-deoxycytidine) is a nucleoside analog and a pyrimidine antimetabolite that is an effective agent for cholangiocarcinoma. The combination of gemcitabine and cisplatin is currently used as the standard of care for locally advanced or metastatic cholangiocarcinoma. However, overall survival and the therapeutic effect remain poor and insufficient [10]. To activate the cytotoxic actions of gemcitabine, cellular uptake and phosphorylation are required to modify gemcitabine to gemcitabine triphosphate (dFdCTP) [11, 12]. dFdCTP is incorporated into replicating DNA, which stalls the replication fork. Then, DNA damage response (DDR) proteins are activated, causing double-strand breaks (DSBs) that exert a cytotoxic effect by blocking DNA synthesis [13–15]. Thus, it is suggested that the nuclear localization of DDR proteins has an important role in gemcitabine sensitivity.

In general, the efficacy of DNA-damaging agents can be reduced by the activities of several DNA repair pathways [16]. First, the DDR histone (H2AX) and phosphoinositol kinase-like kinase [ataxia-telangiectasia mutated (ATM)] are phosphorylated and recruited to DNA DSBs induced by cytotoxic agents, including gemcitabine [17]. The MRE11-RAD50-NBS1 (MRN) complex plays important roles in recognizing and repairing such DNA DSBs. Thus, it is suggested that dysregulation of MRN complex-induced DNA repair contributes to gemcitabine resistance [14, 18]. In fact, it was reported that overexpression of the MRN complex promoted chemoresistance via enhanced DNA repair activity and also inhibited apoptosis [19]. Therefore, the DNA DSB repair machinery regulated by the MRN complex is a strong candidate target for cancer therapies against refractory diseases [16, 20].

The MRN complex may also function during DNA replication in both normal and cancer cells [21, 22]. It has been demonstrated that knockout of any component of the MRN complex causes early embryonic lethality in mice [23–25]. Thus, we focused on karyopherin-α2 (KPNA2), which participates in the nuclear transport of the MRN complex and which is specifically expressed in cancer cells and embryonic stem cells, but not in most normal cells [26, 27]. KPNA2 in cancer cells delivers numerous cargo proteins via nuclear localization signals to the nucleus including the DNA repair-associated MRN complex and proliferation-related protein c-Myc/E2F [28–30]. Moreover, a recent study reported that KPNA2 expression was also associated with the subcellular localization of DDR proteins, such as BRCA1 and RAD51, in breast cancer [18].

Recently, KPNA2 has emerged as a potential biomarker in several cancers [26, 31–38]. Our previous studies identified that high KPNA2 expression was correlated with poor prognosis and cancer progression in esophageal squamous cell carcinoma [35], gastric carcinoma [31], and colorectal carcinoma [39]. Moreover, the KPNA2 suppression strategy induced apoptosis and conferred anti-proliferation activity in several cancer cells [34]. However, the clinical significance of cancer-specific KPNA2 expression and the relationship with the MRN complex and gemcitabine sensitivity in cholangiocarcinoma remain unclear.

The purpose of this study was to identify the function and clinical significance of KPNA2 in cholangiocarcinoma. KPNA2 expression in cholangiocarcinoma tissues was investigated using immunohistochemical analysis to determine whether KPNA2 expression can serve as a prognostic marker of cholangiocarcinoma. Moreover, KPNA2 suppression analysis was performed *in vitro* and *in vivo* to clarify whether KPNA2 may play important roles in proliferation and gemcitabine sensitivity via nuclear translocation of the MRN complex. Our data clarified the significance of KPNA2 expression in clinical cholangiocarcinoma samples and highlighted the possibility of overcoming gemcitabine resistance by targeting KPNA2 in cholangiocarcinoma cells *in vitro* and *in vivo* for the first time.

## RESULTS

### Immunohistochemical expression of KPNA2 in cholangiocarcinoma samples

**Figure 1:**
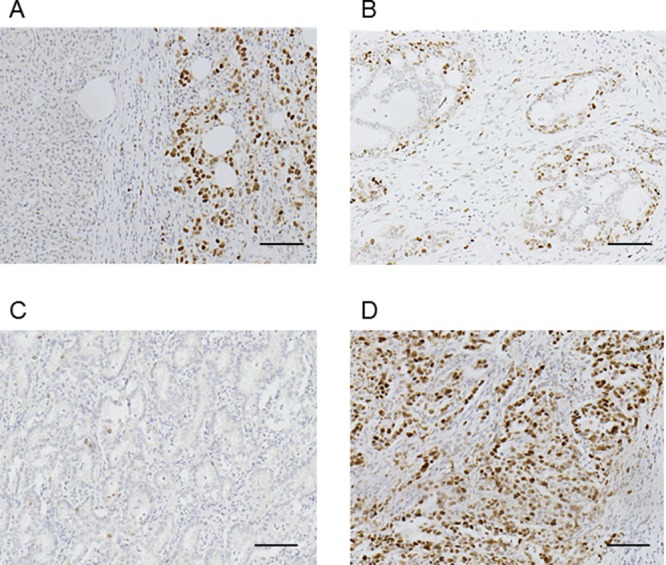
Immunohistochemical staining of karyopherin-α2 (KPNA2) in representative cholangiocarcinoma tissues **A**. KPNA2 expression was mainly localized to the nuclei of tumor cells. Positive expression of nuclear KPNA2 was observed in cancer tissue, but not noncancerous tissue. **B**. Nuclear KPNA2 expression was stronger in marginal regions than in central regions. **C**. Low KPNA2 expression in cholangiocarcinoma. **D**. High KPNA2 expression in cholangiocarcinoma. Original magnification: ×400; scale bar, 100 μm.

KPNA2 expression was higher in the nucleus than in the cytoplasm and higher in malignant tissue than in adjacent noncancerous tissues (Figure 1A). Nuclear KPNA2 expression levels were greater in marginal regions than in central regions (Figure 1B). The MRN complex, which contains representative KPNA2 cargo proteins, was expressed in both noncancerous and cancer tissues (Supplementary Figure 2). Of the 103 patients with cholangiocarcinoma, low KPNA2 expression was found in 23 (22.3%) patients and high expression in 80 (77.7%) (Figure 1C and 1D).

### Association between KPNA2 expression and clinicopathological characteristics

The relationships between clinicopathological parameters and KPNA2 expression in 103 cholangiocarcinoma samples are presented in Table 1. High expression levels of KPNA2 were tightly associated with recurrence in 103 patients with cholangiocarcinoma (*P* = 0.024). There were no significant differences in age, sex, histological type, T factor, tumor size, lymph node metastasis, vascular invasion, perineural invasion, and TNM stage between patients with high and low KPNA2 expression.

**Table 1:** Characteristics of patients with cholangiocarcinoma

Variables	KPNA2 expression		*P*
	Low (*N* = 23)	High (*N* = 80)	
Mean age, y ± SD	66.9 ± 9.0	68.5 ± 8.9	0.513
Sex, *n* (%)			0.414
Male	14 (61)	56 (70)	
Female	9 (39)	24 (30)	
Histological type, *n* (%)			0.215
Well, moderately	19 (83)	56 (70)	
Poor	4 (17)	24 (30)	
T factor (UICC), *n* (%)			0.160
T1, 2	15 (65)	39 (49)	
T3, 4	8 (35)	41 (51)	
Tumor size, *n* (%)			0.978
<30 mm	11 (48)	38 (48)	
≥30 mm	12 (52)	42 (52)	
Lymph node metastasis, *n* (%)			0.062
Absent	18 (78)	46 (58)	
Present	5 (22)	34 (42)	
Vascular invasion, *n* (%)			0.098
Absent	18 (78)	48 (60)	
Present	5 (22)	32 (40)	
Perineural invasion, *n* (%)			0.938
Absent	11 (48)	39 (49)	
Present	12 (52)	41 (51)	
TNM stage (UICC), *n* (%)			0.897
I-II	19 (83)	67 (84)	
III-IV	4 (17)	13 (16)	
Recurrence, *n* (%)			0.024*
Absent	15 (65)	31 (39)	
Present	8 (35)	49 (61)	

UICC: Union for International Cancer Control; JSHBPS: Japanese Society of Hepato-Biliary-Pancreatic Surgery. * = *P* < 0.05.

The relationships between clinicopathological parameters and KPNA2 expression in EHCC or IHCC samples are presented in Supplementary Table 1. KPNA2 expression in EHCC was significantly associated with venous invasion and recurrence (*P* = 0.046 and *P* = 0.025, respectively). High KPNA2 expression in IHCC was associated with lymph node metastasis (*P* = 0.034).

### Association between KPNA2 expression and prognosis

**Figure 2:**
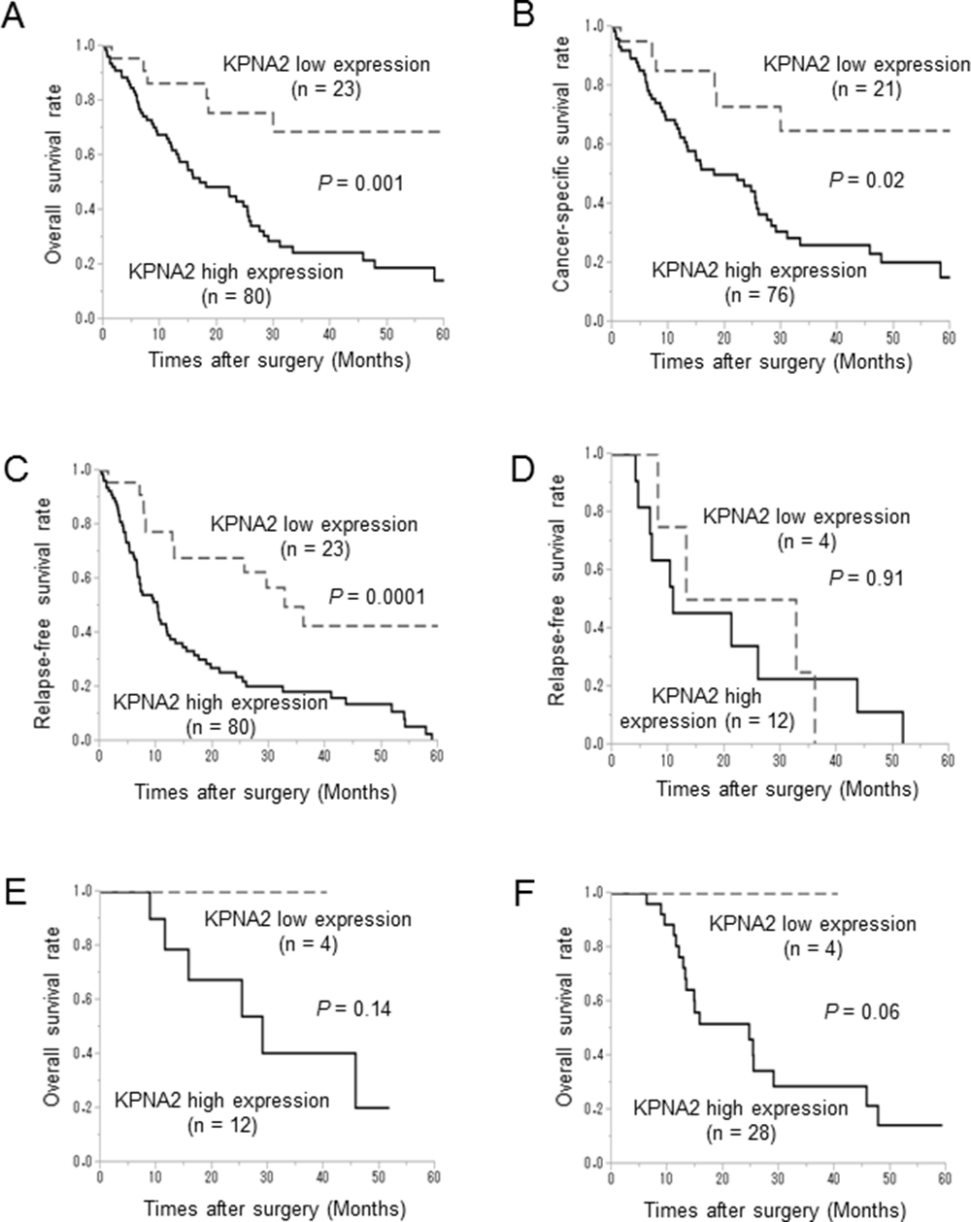
Kaplan–Meier plots showing overall and disease-free survival according to karyopherin-α2 (KPNA2) expression **A**. Among 103 patients with cholangiocarcinoma, overall survival was significantly shorter (*P* = 0.001) among those with high KPNA2 than among those with low expression. **B, C**. High KPNA2 expression was significantly associated with shorter cancer-specific survival (*P* = 0.02) and relapse-free survival (*P* = 0.0001). **D, E**. In 16 patients with cholangiocarcinoma who received gemcitabine as adjuvant chemotherapy after surgical resection, there was no significant difference in relapse-free survival (*P* = 0.91) and overall survival (*P* = 0.14) between those with high and low KPNA2 expression. **F**. In 32 patients with cholangiocarcinoma who received gemcitabine after surgery or recurrence, KPNA2 overexpression tended to be a prognostic indicator of poor overall survival, although there were no significant differences (*P* = 0.06).

The prognostic significance of KPNA2 expression is shown (Figure 2). Patients with high KPNA2 expression exhibited significantly shorter overall survival than those with low KPNA2 expression (*P* = 0.001; Figure 2A). Moreover, KPNA2 overexpression was significantly associated with cancer-specific and relapse-free survival (*P* = 0.02 and *P* = 0.0001, respectively; Figure 2B and 2C).

Next, the potential predictive role of KPNA2 expression during postoperative chemotherapy with gemcitabine was examined. In 16 patients with cholangiocarcinoma who received gemcitabine as an adjuvant chemotherapy, there was no significant difference in relapse-free survival (*P* = 0.91; Figure 2D) or overall survival (*P* = 0.14; Figure 2E) according to KPNA2 expression. In 32 patients with cholangiocarcinoma who received gemcitabine as an adjuvant chemotherapy or to treat recurrence, although KPNA2 overexpression tended to be a prognostic indicator of poor overall survival, there were no statistically significant differences (*P* = 0.06; Figure 2F).

### Univariate and multivariate analysis of clinicopathological factors associated with overall survival in patients with cholangiocarcinoma

Univariate analysis of 103 patients with cholangiocarcinoma revealed that histological type (*P* = 0.015), lymph node metastasis (*P* = 0.035), vascular invasion (*P* = 0.0001), perineural invasion (*P* = 0.017), and KPNA2 expression (*P* = 0.0004) were significantly associated with overall survival. Multivariate analysis revealed that vascular invasion, perineural invasion, and KPNA2 expression [risk ratio (RR) = 3.15; 95% confidence interval (CI) = 1.51–7.54; *P* = 0.001] were independent prognostic indicators of poor survival (Table 2). Multivariate analysis in relation to relapse-free survival revealed that T factor, lymph node metastasis, and KPNA2 expression (RR = 3.29; 95% CI = 1.77–6.75; *P* < 0.0001) were independent predictors of recurrence (Supplementary Table 2). Moreover, KPNA2 overexpression was an independent prognostic indicator of poor survival in 87 patients with EHCC (Supplementary Table 3), and it was significantly associated with overall survival in 16 patients with IHCC (Supplementary Table 4).

**Table 2:** Cox univariate/multivariate regression analysis of variables related to overall survival in patients with cholangiocarcinoma

Clinicopathological variables	Univariate analysis			Multivariate analysis		
	RR	95% CI	*P*	RR	95% CI	*P*
Age (<65 vs. ≥65)	0.82	0.50–1.38	0.453	-	-	-
Sex (male vs. female)	0.79	0.46–1.35	0.406	-	-	-
Histology type (well, moderately vs. poor)	2.07	1.16–3.57	0.015*	1.46	0.80–2.57	0.212
T factor (UICC) (T1, 2 vs. 3, 4)	1.64	0.99–2.74	0.056	-	-	-
Lymph node metastasis (absent vs. present)	1.76	1.04–2.97	0.035*	1.63	0.95–2.80	0.079
Vascular invasion (absent vs. present)	3.07	1.77–5.32	0.0001*	2.26	1.29–3.95	0.005*
Perineural invasion (absent vs. present)	1.83	1.11–3.08	0.017*	2.01	1.19–3.45	0.009*
KPNA2 expression (low vs. high)	3.34	1.65–7.74	0.0004*	3.15	1.51–7.54	0.001*

RR: relative risk; CI: confidence interval; UICC: Union for International Cancer Control. * = *P* < 0.05.

### KPNA2-specific siRNA inhibits tumor cell proliferation and enhances gemcitabine sensitivity *in vitro*

**Figure 3:**
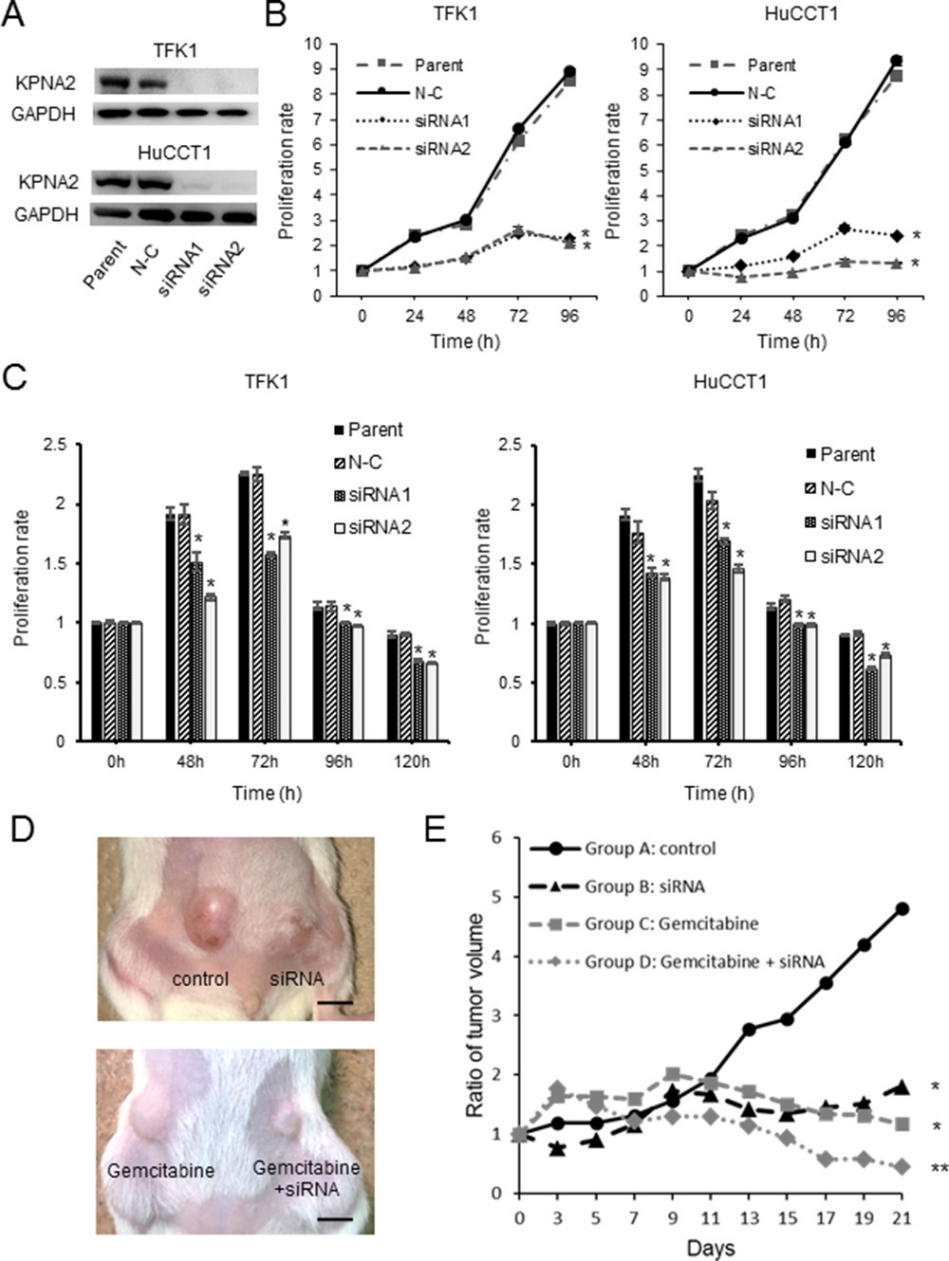
*In vitro* and *in vivo* analysis of karyopherin-α2 (KPNA2) function in cholangiocarcinoma cell lines transfected with KPNA2-specific siRNAs **A**. KPNA2-specific siRNA1 and siRNA2 significantly reduced KPNA2 expression compared with control siRNA in both TFK1 and HuCCT1 cells. **B**. Proliferation of TFK1 and HuCCT1 cells transfected with KPNA2-specific siRNAs was analyzed using the WST assay. The proliferation of KPNA2-depleted cells was significantly lower than that of parent and negative control (N-C)-transfected cells. *P < 0.05 vs. N-C transfected cells. **C**. Proliferation and gemcitabine sensitivity of TFK1 and HuCCT1 cells transfected with KPNA2 siRNAs were analyzed using the WST assay. Gemcitabine sensitivity in KPNA2 siRNA-treated cells was enhanced compared with that in control cells for both TFK1 and HuCCT1 cells. *P < 0.05 vs. N-C transfected cells. **D**. Representative pictures of subcutaneous TFK1-xenograft tumors after 3 weeks of gemcitabine treatment. KPNA2 siRNA (siRNA group and gemcitabine + siRNA group) and phosphate-buffered saline (PBS) (control group and gemcitabine group) were injected into tumors of the right and left flanks, respectively. Scale bar, 5 mm. **E**. The tumor growth curve of gemcitabine-treated TFK1-xenograft tumors with or without KPNA2 siRNA local injection. *P < 0.05 siRNA group, the gemcitabine group vs. control group. **P < 0.05, the gemcitabine + siRNA group vs. siRNA group, gemcitabine group.

KPNA2 was highly expressed in TFK1 and HuCCT1 cells. Both KPNA2-specific siRNAs significantly reduced KPNA2 levels, as compared with control siRNA (Figure 3A). The proliferation of KPNA2-depleted cells was significantly lower than that of parent and negative control-transfected TFK1 and HuCCT1 cells (Figure 3B). Gemcitabine sensitivity was enhanced in KPNA2 siRNA-treated cells compared with control TFK1 and HuCCT1 cells (Figure 3C).

### KPNA2-specific siRNA inhibits tumor cell proliferation and enhances gemcitabine sensitivity *in vivo*

Mice with xenografted cholangiocarcinoma tumors were treated with KPNA2 siRNA and/or gemcitabine. The volume of tumors injected with KPNA2 siRNA significantly decreased compared with that of control tumors (Figure 3D). The volume of gemcitabine-treated tumors injected with KPNA2 siRNA significantly decreased, as compared with tumors treated with only gemcitabine (Figure 3D). The tumor growth rate was measured and calculated at each time point (Figure 3E). In the control group, the tumor volume significantly increased after 3 weeks. On the contrary, tumor growth decreased in mice treated with KPNA2 siRNA. Moreover, tumor growth was further decreased in mice treated with KPNA2 siRNA and gemcitabine compared with that in mice treated with gemcitabine or KPNA2 siRNA only.

### Expression of KPNA2 and the MRN complex in TFK1 cells and clinical cholangiocarcinoma tissues

**Figure 4:**
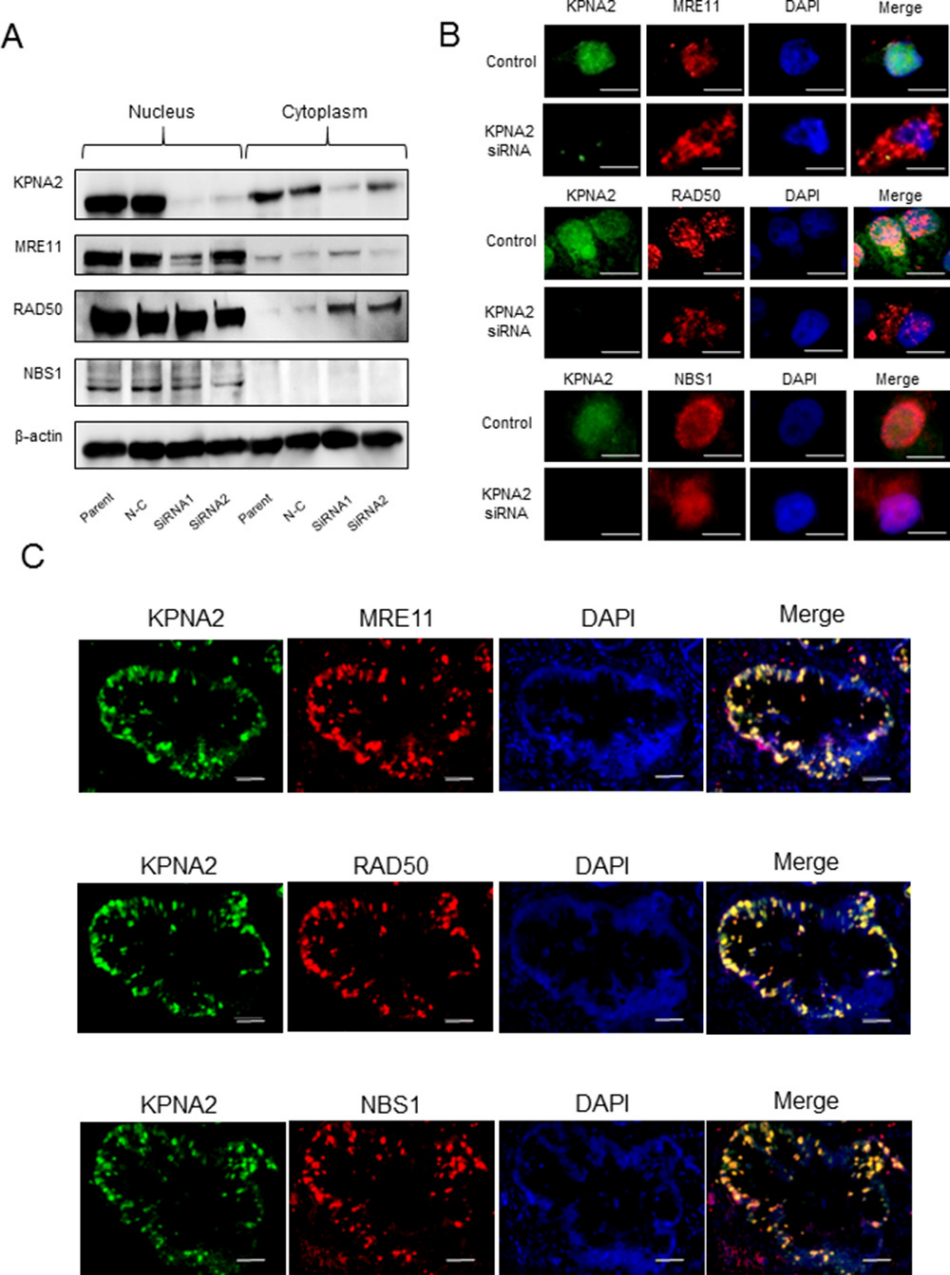
Karyopherin-α2 (KPNA2) and MRE11-RAD50-NBS1 (MRN) complex expression in cholangiocarcinoma cell lines and tissues **A**. TFK1 cells were transfected with KPNA2-specific siRNA, and the protein expression of KPNA2 and the MRN complex in both the nucleus and cytoplasm was analyzed using Western blotting. β-actin was used as a loading control. **B**. Intracellular localization of KPNA2 and the MRN complex in TFK1 cells treated with KPNA2 or control siRNA. TFK1 cells were immunostained with antibodies against KPNA2 (green) and MRE11, RAD50, and NBS1 (red). All sections were counterstained with 4′, 6-diamidino-2-phenylindole (DAPI, blue). Original magnification: ×600; scale bar, 20 μm. **C**. Intracellular localization of KPNA2 and the MRN complex in clinical cholangiocarcinoma tissue. Representative cholangiocarcinoma tissue was immunostained with antibodies against KPNA2 (green) and MRE11, RAD50, and NBS1 (red). All sections were counterstained with DAPI (blue). Original magnification: ×400; scale bar, 100 μm.

Protein expression of the MRN complex was decreased in the nuclei of KPNA2-depleted cells. By contrast, protein expression in the cytoplasm was increased in both MRE11 and RAD50 cells (Figure 4A). Immunocytochemical analysis illustrated that expression of MRE11, RAD50, and NBS1 was mainly localized to the nucleus, and these proteins were colocalized with KPNA2 in control cells. As observed in Western blot analysis of KPNA2 siRNA-treated cells, expression of the MRN complex was decreased in the nucleus and increased in the cytoplasm (Figure 4B). Moreover, colocalization of KPNA2 and the MRN complex was observed in the nuclei of cells in clinical cholangiocarcinoma tissues (Figure 4C).

## DISCUSSION

In this study, overexpression of nuclear KPNA2 was significantly associated with poor prognosis and chemosensitivity of cholangiocarcinoma. KPNA2 overexpression was an independent prognostic factor of overall and relapse-free survival. Depletion of KPNA2 decreased the viability of gemcitabine-treated cholangiocarcinoma cells *in vitro* and *in vivo*. Expression of the MRN complex was colocalized with that of KPNA2 in both cholangiocarcinoma tissues and cell lines. These findings clarified a new regulatory mechanism of gemcitabine sensitivity in cholangiocarcinoma by targeting the nuclear–cytoplasmic transportation of the MRN complex via KPNA2.

KPNA2 overexpression has been reported in several malignant tumors [26]. Several studies revealed that high KPNA2 expression is associated with cancer progression and poor prognosis [31, 33, 35, 38, 41–46], suggesting KPNA2 as a potential prognostic marker. In the current study, nuclear KPNA2 expression exhibited a significant association with poor prognosis in cholangiocarcinoma. Furthermore, a significant correlation was observed between high KPNA2 expression and shorter overall survival and relapse-free survival in patients with EHCC and IHCC. The significance of KPNA2 expression in cholangiocarcinoma is assumed to be consistent with the findings of previous reports. The present study results indicated that KPNA2 expression may be an independent prognostic marker of cholangiocarcinoma.

Several studies have reported that KPNA2 plays important roles in cell proliferation [33, 37] and that KPNA2 overexpression is associated with increased cell proliferation, while depletion of KPNA2 inhibits proliferation and increased apoptosis [31, 34]. In the present study, siRNA approaches were employed for KPNA2 functional analysis *in vitro* and *in vivo* because KPNA2 was expressed in all cholangiocarcinoma, gastric cancer, and colon cancer cell lines in our department, and an association between KPNA2 suppression and cell proliferation was noted in cholangiocarcinoma cells. KPNA2 is an adaptor protein that mediates the nuclear import of various proteins associated with cancer proliferation. Previous reports demonstrated that cell cycle-related transcription factors, such as E2F and c-Myc, were transferred from the cytoplasm to the nucleus by KPNA2 [44, 47]. These transcription factors may be involved in the molecular mechanism underlying reduced proliferation upon KPNA2 depletion in cholangiocarcinoma. Our results suggest a critical role of KPNA2 in the proliferation of cholangiocarcinoma cells.

In the present study, KPNA2 was significantly associated with survival, despite the fact that there were no correlations with the well-known prognostic factors, i.e., histological type and lymph node metastasis. On the other hand, low KPNA2 expression tended to be associated with a favorable outcome of gemcitabine treatment. In addition, gemcitabine sensitivity was enhanced in KPNA2-depleted cells *in vitro* and *in vivo*. Gemcitabine is a pyrimidine nucleoside analog that exhibits cytotoxic activity only after entry into the cell and phosphorylation [48]. dFdCTP is incorporated into DNA and causes stalling of replication forks and DNA DSBs [13–15]. The DNA repair mechanisms for the removal of nucleotide analogs from DNA are currently not well understood. Ewald *et al*. investigated the role of the MRN complex and ATM in response to stalled replication forks caused by gemcitabine and found that ATM, MRE11, and RAD50, but not NBS1 or H2AX, contributed to cell survival and recovery from gemcitabine-induced replication fork stalling [14]. DNA repair mechanisms are generally considered strong targets in cancer therapy [16, 20]. However, the MRN complex also plays an important role in normal DNA replication [21, 22]. In the present study, KPNA2 expression was high in malignant tissues, but rare in adjacent normal tissues. In contrast, MRN complex expression was high in both normal and cancer tissues (Supplementary Figure 2). These data clearly illustrated that the MRN complex was colocalized with KPNA2 in cholangiocarcinoma cells, but not in the surrounding normal cells (Figure 4C). Moreover, the nuclear–cytoplasmic transportation of the MRN complex could be regulated using KPNA2 siRNAs. These data suggest that targeting cancer-specific KPNA2 and not directly targeting the MRN complex, which is expressed in normal cells, may suppress the DNA repair pathways mediated by the MRN complex, thereby enhancing gemcitabine sensitivity and improving prognosis of cholangiocarcinoma.

In conclusion, the results of this study demonstrated that KPNA2 expression was significantly associated with poor prognosis in patients with cholangiocarcinoma. In addition, KPNA2 regulated cell proliferation and gemcitabine sensitivity *in vitro* and *in vivo*. Furthermore, expression of the MRN complex was colocalized with that of KPNA2 in both cholangiocarcinoma tissues and cell lines, and cellular localization of the MRN complex was controlled by KPNA2 regulation. The nuclear MRN complex, which is transferred by KPNA2, has an important role in gemcitabine sensitivity. These results suggest that KPNA2 may be a useful prognostic marker and an effective therapeutic target for cholangiocarcinoma.

## MATERIALS AND METHODS

### Patients and samples

The 103 surgically resected cholangiocarcinoma samples [87 EHCC and 16 intrahepatic cholangiocarcinoma (IHCC)] were obtained from patients who underwent surgical treatment at the Department of General Surgical Science, Gunma University Hospital, and Saiseikai Maebashi Hospital between 1995 and 2011. No patient received chemotherapy or irradiation prior to surgery. In 18 cases, there was a microscopic tumor at the cutting margin (R1-resection). Of 54 patients (52%) who received adjuvant chemotherapy, 13 received gemcitabine (Gemzar; Eli Lilly and Company, Indianapolis, IN, USA), 23 received S-1 (TS-1; Taiho Pharmaceutical, Tokyo, Japan); 3 received gemcitabine + S-1, and 15 received tegafur–uracil (Taiho Pharmaceutical). Six patients (6%) received adjuvant irradiation. A total of 32 patients with cholangiocarcinoma received gemcitabine as adjuvant chemotherapy or to treat recurrence. All clinical samples were used in accordance with institutional guidelines and the Helsinki Declaration after obtaining written informed consent from all participants. Tumor stages were classified according to the seventh tumor-node-metastasis (TNM) classification of the Union for International Cancer Control (UICC) and the sixth Classification of Biliary Tract Carcinoma of the Japanese Society of Hepato-Biliary-Pancreatic Surgery (JSHBPS). Clinicopathological findings were based on clinical records and pathology reports.

### Immunohistochemical analysis

The resected surgical specimens were fixed with 10% formaldehyde, embedded in paraffin blocks, cut into 3-μm-thick sections, and mounted onto glass slides. All sections were incubated at 60°C for 60 min, deparaffinized in xylene, rehydrated, and incubated with fresh 0.3% hydrogen peroxide in 100% methanol for 30 min at room temperature to block endogenous peroxidase activity. After rehydration with a graded series of ethanol, the sections were then heated in boiled water and Immunosaver (Nisshin EM, Tokyo, Japan) at 98°C for 45 min. Nonspecific binding sites were blocked by incubation with Protein Block Serum-Free (DAKO, Carpentaria, CA, USA) for 30 min. The sections were then incubated with rabbit anti-KPNA2 polyclonal antibody (1:400; ab84440, anti-KPNA2 antibody, Abcam, Cambridge, England), anti-MRE11 rabbit monoclonal antibody (1:400; ab109623, anti-Mre11 antibody, Abcam), anti-RAD50 mouse monoclonal antibody (1:400; ab89, anti-Rad50 antibody, Abcam), or anti-p95 NBS1 rabbit monoclonal antibody (1:400; ab32074, anti-p95 NBS1 antibody, Abcam) in Can Get Signal^®^ immunostain (Toyobo Co., Ltd., Osaka, Japan) overnight at 4°C and at room temperature for 30 min. The sections were washed in phosphate-buffered saline (PBS) and incubated with Histofine Simple Stain MAX-PO (MULTI) (Nichirei Co., Tokyo, Japan) for 45 min at room temperature.

The chromogen 3,3′-diaminobenzidine tetrahydrochloride was applied as a 0.02% solution containing 0.005% H_2_O_2_ in 50 nM ammonium acetate–citrate acid buffer (pH 6.0). The sections were lightly counterstained with Mayer's hematoxylin and mounted. No detectable staining was evident of negative controls established by omitting the primary antibody. Gastric cancer samples, which were regarded to have high-level expression in a previous study [31], were used as positive controls.

### Evaluation of immunostaining

Immunohistochemical staining results for KPNA2 were evaluated as previously described [31]. Immunohistochemical slides were scanned and evaluated by two independent researchers in a blinded manner. The intensity of nuclear KPNA2 staining was scored as follows: 0, no staining; 1+, weak staining; 2+, moderate staining; and 3+, strong staining. The percentage of cells with nuclear staining was calculated by examining at least 1,000 cancer cells in five representative areas. The nuclear KPNA2 staining percentages were scored as follows: 0, no staining; 1+, 1%–10%; 2+, 11%–50%; and 3+, 51%–100%. The score was defined as the percentages multiplied by the intensity score (0, 1+, 2+, 3+, 4+, 6+, or 9+) (Supplementary Figure 1). The cut-off point was defined as follows: scores of 0–3 were considered to indicate low expression, whereas scores of 4, 6, and 9 indicated high expression.

### Cell culture

The human cholangiocarcinoma cell lines TFK1 and HuCCT1 were obtained from RIKEN BRC (Bio-Resource Center) through the National Bio-Resource Project of the MEXT, Japan, in 2008. TFK1 and HuCCT1 cell lines were authenticated by short tandem repeat DNA profiling by BEX Co., Ltd. (Tokyo, Japan) in 2015. These cells were cultured in RPMI 1640 medium (Wako, Osaka, Japan) containing 10% Fetal Bovine Serum (FBS) and supplemented with 100 U/ml of penicillin and streptomycin sulfate (Invitrogen, Carlsbad, CA, USA). Cells were maintained at 37°C in a humidified 5% CO_2_ incubator and collected with TrypLE™ Express Cell Dissociation Enzyme (Thermo Fisher Scientific, Kanagawa, Japan).

### *In vitro* transfection of KPNA2-specific siRNA

KPNA2-specific siRNAs (siRNA 1: ACCUGCUGGGCUAUUUCCUACCUUA, UAAGGUAGGAAAUAGCCCAGCAGGU, siRNA 2: CAGAUACCUGCUGGGCUAUUUCCUA, UAGGAAAUAGCCCAGCAGGUAUCUG) and negative-control siRNA were purchased from GeneDesign, Inc. (Osaka, Japan). TFK1 and HuCCT1 cells were transfected with control or KPNA2-specific siRNA using Lipofectamine RNAiMAX regents (Invitrogen) according to the manufacturer's recommendation. One day before transfection, TFK1 and HuCCT1 cells were seeded at 2 × 10^5^ cells/well in 2 ml of medium (RPMI 1640 with 10% FBS and 1% antibiotic) in 6-well flat-bottomed plates. After 24 h of incubation, cells were washed twice with PBS and Opti-MEM I Reduced Serum Medium (Invitrogen). The complexes of KPNA2 siRNA and Lipofectamine RNAiMAX (1 ml) were added to each well containing cells. After 6 h of incubation, 1 ml each of 20% FBS and antibiotic-free RPMI 1640 were added to each well. The cells were transfected with KPNA2 siRNAs again at 96 h of incubation after the first transfection to strongly silence KPNA2 protein expression as previously described [34].

### Protein extraction and western blot analysis

Western blot analysis was used to confirm KPNA2 protein expression in cholangiocarcinoma cell lines. Total proteins, nucleoproteins, and cytoplasmic proteins were extracted from TFK1 and HuCCT1 cells using the PRO-PREP Protein Extraction Solution Kit (iNtRON Biotechnology, Sungnam, Kyungki-Do, Korea) and the NE-PER Nuclear and Cytoplasmic Extraction Kit (Thermo Scientific). The proteins were separated using SDS–PAGE with 10% Bis–Tris gels and transferred to membranes. The membranes were blocked with 5% skim milk and incubated overnight at 4°C with anti-KPNA2 rabbit polyclonal antibody (1:1000; Abcam), anti-MRE11 rabbit monoclonal antibody (1:2000; Abcam), anti-RAD50 mouse monoclonal antibody (1:2000; Abcam), anti-p95 NBS1 rabbit monoclonal antibody (1:2000; Abcam), GAPDH antibody (1:1000; Santa Cruz Biotechnology, Santa Cruz, CA, USA), or anti-β-actin mouse monoclonal antibody (1:1000; clone AC-74; Sigma, St. Louis, MO, USA). The membranes were then treated with horseradish peroxidase (HRP)-conjugated secondary antibodies. Specific signals were detected using the ECL Prime Western Blotting Detection System (GE Healthcare, Tokyo, Japan) and quantified using an Image Quant LAS 4000 instrument (GE Healthcare).

### *In vitro* proliferation assay

Cell proliferation analysis of siRNA-transfected cultures was performed using TFK1 and HuCCT1 cells. These cells were cultured in 96-well culture plates, at 10,000 cells/well, in 100 μl of medium. After initial cell seeding, cell viability was analyzed using the Cell Counting Kit-8 (Dojindo Laboratories, Kumamoto, Japan). Evaluations were performed after 0, 24, 48, 72, and 96 h. The cell counting solution was added at a concentration of 10 μl/well, and cells were incubated at 37°C in a humidified 5% CO_2_ atmosphere for 2 h 30 min. The optical density of the wells was measured at 450 nm using an xMarkTM Microplate Absorbance Spectrophotometer (Bio-Rad Laboratories, Hercules, CA, USA). All results were derived from 12 sets of duplicated experiments.

### Gemcitabine sensitivity assay

TFK1 and HuCCT1 cells transfected with KPNA2 siRNAs were dispensed into 96-well plates. Twenty-four hours after the initial seeding, gemcitabine was added at a final concentration of 10 μM. Evaluations were performed after 0, 48, 72, 96, and 120 h. To quantitate cell viability using the Cell Counting Kit-8 (Dojindo Laboratories), 10 μl/well of the cell counting solution was added and the plates were incubated at 37°C in a humidified 5% CO2 atmosphere for 2 h 30 min. The optical density of the wells was measured at 450 nm using an xMarkTM Microplate Absorbance Spectrophotometer. All results were derived from 12 sets of duplicated experiments.

### Xenograft mouse models

Mouse experiments were performed in compliance with the guidelines of the Institute for Laboratory Animal Research at Gunma University, Maebashi, Japan. In total, a TFK1 cell suspension (3 × 10^6^ cells in 200 μl of PBS) was subcutaneously (s.c.) injected into the bilateral flanks of 8-week-old female NOD-SCID mice (CLEA Japan, Inc, Tokyo, Japan).

### Treatment with KPNA2 siRNA and gemcitabine *in vivo*

Mice with s.c. xenografted tumors were treated with KPNA2 siRNA and/or gemcitabine when the tumor reached a maximum diameter of 5 mm. Mice were randomized into four groups as follows: group A, control; group B, KPNA2 siRNA alone; group C, gemcitabine alone; and group D, KPNA2 siRNA + gemcitabine. Each group contained three mice (n = 3).

*In vivo* silencing of KPNA2 was performed as described by Takei *et al* [40]. Mice were deeply anesthetized with pentobarbital (40 mg/kg intraperitoneal injection). A fork-type electrode was inserted into the tumor, scooping from the bottom of the tumor, and then KPNA2 siRNA (2000 pmol/100 μl) or PBS (100 μl) was injected into each. Immediately, the plate-type electrode was put in contact with the surface of the tumor and electric pulses were delivered to each tumor using the CUY21EDIT II Next-Generation Electroporator (Bex, Japan). In groups A and C, PBS was injected into tumors in the bilateral flanks. In group B and D, KPNA2 siRNA was injected into tumors of the right flanks and PBS was injected into tumors of the left flanks. After injection of the siRNA and electroporation, gemcitabine was administered via intraperitoneal injection as follows: group A and B, 400 μl of PBS; and groups C and D, 1 mg gemcitabine/PBS 200 μl/10 g mouse body weight. The body weights of the mice were measured every other day (Supplementary Figure 3). Tumor diameters were measured every other day and calculated using the following formula: tumor volume = S × S × L/2, where S is the short diameter of the tumor in millimeters and L is the maximum diameter of the tumor in millimeters. Therapy was repeated every 5 days for 3 weeks. The mice were then sacrificed and the tumors were removed. These xenografted tumor formations were microscopically validated after hematoxylin and eosin staining.

### Fluorescent immunocytochemistry

Immunofluorescence of KPNA2 siRNA-transfected cultures of TFK1 cells was performed. Forty-eight hours after transfection according to the aforementioned method, the cells were fixed with 4% paraformaldehyde for 20 min at room temperature and then incubated with PBS containing 0.01% Triton X-100 for 15 min at room temperature. After inhibiting endogenous peroxidase activity, nonspecific binding sites were blocked with TNT (Tris-NaCl-Tween) blocking buffer for 30 min. This was followed by incubation with primary antibodies against KPNA2 (1:400) for 1 h at room temperature. The sections were then rinsed in TNT buffer three times for 5 min each and incubated for 30 min at room temperature with HRP-conjugated secondary antibody. After washing with TNT buffer three times for 5 min each, fluorescein with the tyramide signal amplification (TSA) method (Opal™ 3-Plex Kit; Perkin Elmer, Waltham, MA, USA) was applied to the tissue section for 10 min. The sections were then washed in TNT buffer three times for 5 min each, and the secondary detection reagents were added via similar methods [Cy3-conjugated streptavidin; secondary antibodies: MRE11 (1:800), RAD50 (1:800), and NBS1 (1:800)]. All sections were counterstained with 4′, 6-diamidino-2-phenylindole (DAPI) and examined under an All-in-One BZ-X710 fluorescence microscope (Keyence Corporation).

### Fluorescent immunohistochemical analysis

The resected surgical cholangiocarcinoma specimens were fixed using 10% formaldehyde, embedded in paraffin blocks, cut into 3-μm-thick sections, and mounted onto glass slides. Fluorescent immunohistochemical analysis was performed using TSA methods according to the manufacturer's protocol (Opal™ 3-Plex Kit). Following deparaffinization, endogenous peroxidase was inhibited by incubating sections in 0.3% H_2_O_2_ in methanol for 30 min. After washing in ethanol and PBS, the sections were boiled in citrate buffer (pH 6.4) for 15 min in a microwave. Nonspecific binding sites were blocked by incubation with TNT blocking buffer for 30 min and the sections were incubated with the primary antibodies against KPNA2 (1:600) for 1 h at room temperature. The sections were rinsed in TNT buffer three times for 5 min each and then incubated for 30 min at room temperature with HRP-conjugated secondary antibody. After washing with TNT buffer three times for 5 min each, fluorescein with TSA methods (Opal™ 3-Plex Kit) was applied to the tissue section for 10 min. The sections were then washed in TNT buffer three times for 5 min each and the application of secondary detection reagents was performed using similar methods [Cy3-conjugated streptavidin; secondary antibodies: MRE11 (1:800), RAD50 (1:800), and NBS1 (1:800)]. All sections were counterstained using DAPI and examined under an All-in-One BZ-X710 fluorescence microscope.

### Statistical analysis

Statistical significance was analyzed using the Mann–Whitney U test or ANOVA for continuous variables and the chi-squared test or Fisher's extract test for categorical variables. Survival curves were calculated using the Kaplan–Meier method. Differences between survival curves were analyzed using the log-rank test. Prognostic factors were examined by univariate and multivariate analyses using Cox's proportional hazard model. Results were considered statistically significant when the relevant *P* value was <0.05, and all statistical analyses were performed using JMP 11 software (SAS Institute, Cary, NC, USA).

## SUPPLEMENTARY MATERIALS FIGURES AND TABLES




