# Supplementary material for: Overexpression of karyopherin-α2 in cholangiocarcinoma correlates with poor prognosis and gemcitabine sensitivity via nuclear translocation of DNA repair proteins
Source: Oncotarget. 2017 Feb 2;8(26):42159–72. doi: 10.18632/oncotarget.15020 (PMC5522057; doi:10.18632/oncotarget.15020)
Supplement: Supplementary file 1 [file oncotarget-08-42159-s001.pdf]

# Overexpression of karyopherin-α2 in cholangiocarcinoma correlates with poor prognosis and gemcitabine sensitivity via nuclear translocation of DNA repair proteins

## SUPPLEMENTARY MATERIALS

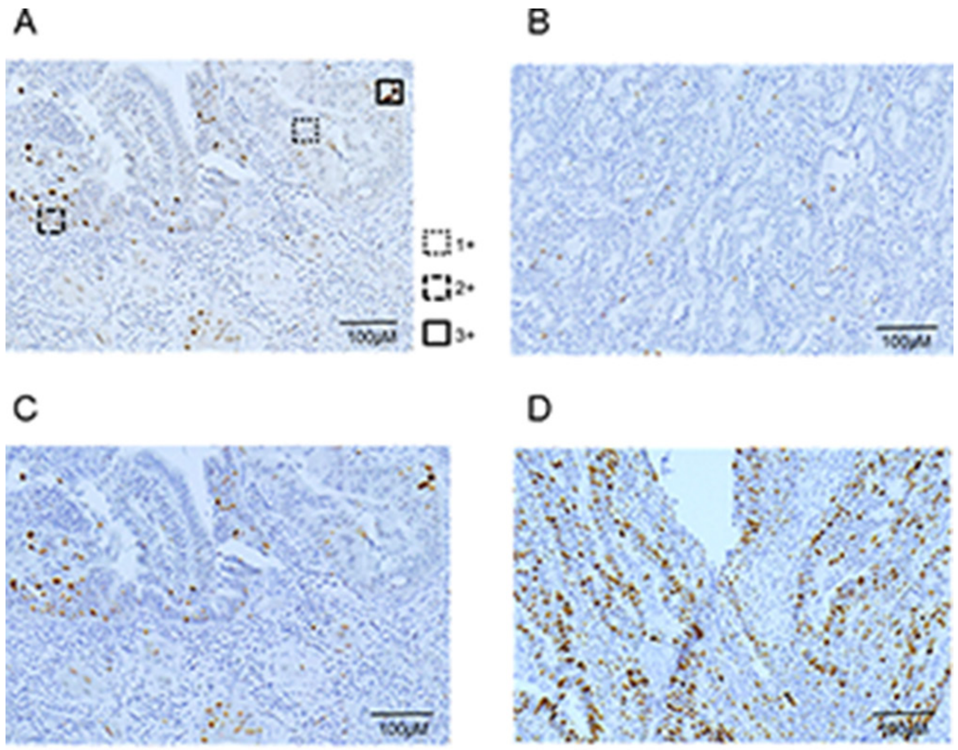


E

| intensity \ proportion | 0<br>0% | 1+<br>(1%-10%) | 2+<br>(11%-50%) | 3+<br>(50% <) |
|---|---|---|---|---|
| 0 | 0 | 0 | 0 | 0 |
| 1+ | 0 | 1+ | 2+ | 3+ |
| 2+ | 0 | 2+ | 4+ | 6+ |
| 3+ | 0 | 3+ | 6+ | 9+ |

KPNA2 low expression group: 0+, 1+, 2+, 3+
KPNA2 high expression group: 4+, 6+, 9+

**Supplementary Figure 1: Immunohistochemical expression of karyopherin-α2 (KPNA2) in cholangiocarcinoma tissues.** **A.** Positive KPNA2 expression was mainly localized to the nuclei of tumor cells. Nuclear KPNA2 expression was evaluated according to staining intensity and scored as follows: 0, no staining; 1+, weak staining; 2+, moderate staining; and 3+, strong staining. **B, C, D.** The nuclear KPNA2 staining percentages were scored as follows: 0, no staining; 1+, 1%–10% (B); 2+, 11%–50% (C); and 3+, 51%–100% (D). **E.** The score was defined as the percentage score multiplied by the intensity score. The cut-off point was defined as follows: grades 0–3 were considered low expression, whereas grades 4, 6, and 9 were considered high expression.

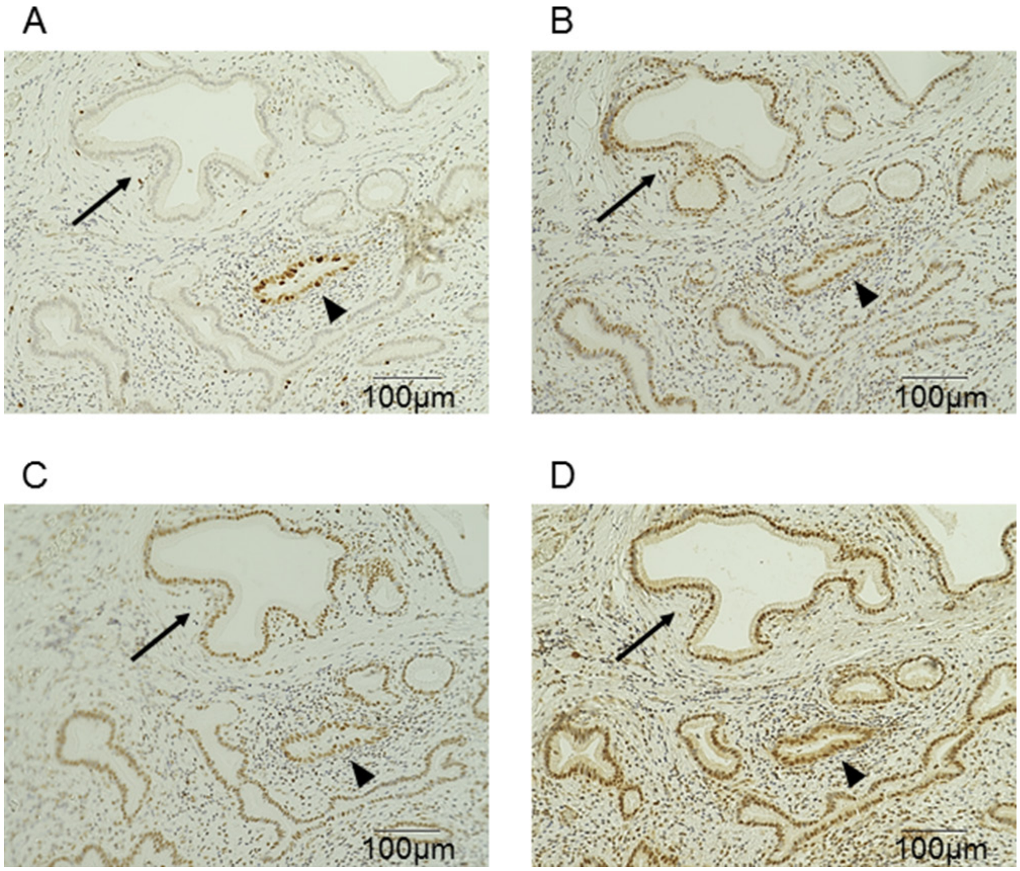


**Supplementary Figure 2: Immunohistochemical expression of karyopherin-α2 (KPNA2) and the MRE11-RAD50-NBS1 (MRN) complex in a representative cholangiocarcinoma tissue including noncancerous cells. A.** Positive KPNA2 expression was mainly restricted to cancer tissues (arrowhead), whereas it was rare in noncancerous tissues (arrow) (A). **B, C, D.** Representative KPNA2 cargo proteins MRE11 (B), RAD50 (C), and NBS1 (D) were expressed in both noncancerous (arrow) and cancer tissues (arrowhead).

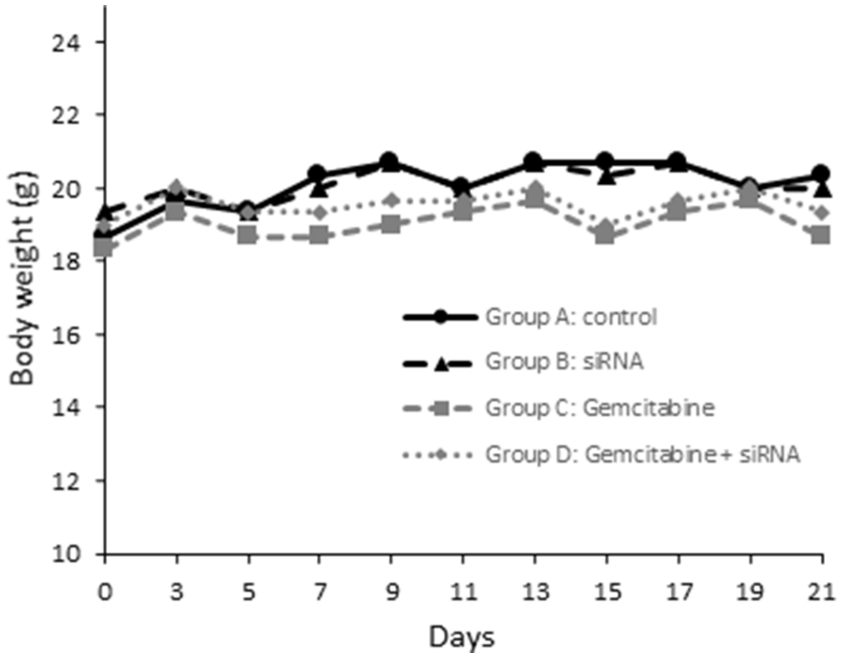


**Supplementary Figure 3: The relationship between mice body weight and treatments in the xenograft experiments.** The body weights of the mice were measured every other day. There was no correlation between the body weight and treatments.

**Supplementary Table 1: Characteristics of patients with extrahepatic cholangiocarcinoma and intrahepatic cholangiocarcinoma**

See Supplementary File 1

**Supplementary Table 2: Cox univariate/multivariate regression analysis of variables related to relapse-free survival in patients with cholangiocarcinoma**

| Clinicopathological variables | Univariate analysis | | | Multivariate analysis | | |
|---|---|---|---|---|---|---|
| | **RR** | **95% CI** | ***P*** | **RR** | **95% CI** | ***P*** |
| Age (<65 vs. ≥65) | 0.85 | 0.55–1.34 | 0.472 | - | - | - |
| Sex (male vs. female) | 0.62 | 0.37–1.00 | 0.052 | - | - | - |
| Histological type (well, moderately vs. poor) | 1.89 | 1.14–3.05 | 0.015* | 1.50 | 0.89–2.45 | 0.123 |
| T factor (UICC) (T1, 2 vs. 3, 4) | 1.73 | 1.12–2.70 | 0.014* | 1.62 | 1.02–2.57 | 0.041* |
| Lymph node metastasis (absent vs. present) | 2.40 | 1.48–3.89 | 0.0005* | 2.09 | 1.27–3.46 | 0.004* |
| KPNA2 expression (low vs. high) | 3.41 | 1.85–6.93 | <0.0001* | 3.29 | 1.77–6.75 | <0.0001* |

RR: relative risk; CI: confidence interval; UICC: Union for International Cancer Control. * = $P < 0.05$.

**Supplementary Table 3: Cox univariate/multivariate regression analysis of variables related to overall survival in patients with extrahepatic cholangiocarcinoma**

| Clinicopathologic variable | Univariate analysis | | | Multivariate analysis | | |
|---|---|---|---|---|---|---|
| | RR | 95% CI | *P* | RR | 95% CI | *P* |
| Age (<65 vs. ≧65) | 0.82 | 0.48–1.40 | 0.465 | – | – | – |
| Gender (male vs. female) | 0.81 | 0.44–1.42 | 0.473 | – | – | – |
| Histology type (well, moderately vs. poor) | 2.18 | 1.21–3.82 | 0.011* | 2.07 | 1.11-3.76 | 0.023* |
| T factor (T1, 2 vs. 3, 4) | 1.77 | 1.04–3.07 | 0.037* | 1.05 | 0.57-1.95 | 0.879 |
| Lymph node metastasis (absent vs. present) | 1.68 | 0.96–2.91 | 0.069 | – | – | – |
| Lymphatic invasion (0, 1 vs. 2, 3) | 2.67 | 1.49–4.79 | 0.001* | 2.14 | 1.13-4.02 | 0.019* |
| Venous invasion (0, 1 vs. 2, 3) | 3.14 | 1.75–5.60 | 0.0002* | 1.54 | 0.78-3.06 | 0.211 |
| Perineural invasion (0, 1 vs. 2, 3) | 2.32 | 1.24–4.73 | 0.007* | 2.03 | 1.06-4.24 | 0.032* |
| KPNA2 expression (low vs. high) | 3.01 | 1.37–7.98 | 0.004* | 2.64 | 1.18-7.15 | 0.017* |

RR: relative risk; CI: confidence interval. * = *P*-value < 0.05.

**Supplementary Table 4: Cox Univariate regression analysis of variables related to overall survival in patients with intrahepatic cholangiocarcinoma**

| Clinicopathologic variable | Univariate analysis | | |
|---|---|---|---|
| | **RR** | **95% CI** | ***P*** |
| Age (<65 vs. ≧65) | 1.22 | 0.19–23.5 | 0.851 |
| Gender (male vs. female) | 0.78 | 0.14–4.27 | 0.766 |
| Tumor size (<50mm vs. ≧50) | 0.63 | 0.09–3.23 | 0.583 |
| Vascular invasion (absent vs. present) | 3.77 | 0.70–28.21 | 0.123 |
| T factor (T1, 2 vs. 3, 4) | 0.45 | 0.02–2.93 | 0.440 |
| Lymph node metastasis (absent vs. present) | 2.24 | 0.41–12.29 | 0.334 |
| KPNA2 expression (low vs. high) | 8.13 | 1.26–158.32 | 0.026* |

RR: relative risk; CI: confidence interval. * = $P$-value < 0.05.
