# Supplementary material for: Overexpression of karyopherin-α2 in cholangiocarcinoma correlates with poor prognosis and gemcitabine sensitivity via nuclear translocation of DNA repair proteins
Source: Oncotarget. 2017 Feb 2;8(26):42159–72. doi: 10.18632/oncotarget.15020 (PMC5522057; doi:10.18632/oncotarget.15020)
Supplement: Supplementary file 2 [file oncotarget-08-42159-s002.docx]

**Supplementary Table 1: Characteristics of patients with extrahepatic cholangiocarcinoma and intrahepatic cholangiocarcinoma**

| Extrahepatic cholangiocarcinoma (*N* = 87) | | | |  | Intrahepatic cholangiocarcinoma (*N* = 16) | | | |
| --- | --- | --- | --- | --- | --- | --- | --- | --- |
| Factors | KPNA2 expression | | |  | Factors | KPNA2 expression | | |
|  | Low (*N* = 16) | High (*N* = 71) | *P* |  |  | Low (*N* = 7) | High (*N* = 9) | *P* |
| Mean age, years ± SD | 65.1 ± 9.5 | 68.4 ± 9.2 | 0.228 |  | Mean age, years ± SD | 70.9 ± 7.7 | 69.4 ± 6.9 | 0.632 |
| Sex, *n* (%) |  |  | 0.469 |  | Sex, *n* (%) |  |  | 1.000 |
| Male | 10 (63) | 51 (72) |  |  | Male | 4 (57) | 5 (56) |  |
| Female | 6 (37) | 20 (28) |  |  | Female | 3 (43) | 4 (44) |  |
| Histology type, *n* (%) |  |  | 0.312 |  | Histology type, *n* (%) |  |  | 1.000 |
| Well, moderately | 13 (81) | 49 (69) |  |  | Well, moderately | 6 (86) | 7 (78) |  |
| Poor | 3 (19) | 22 (31) |  |  | Poor | 1 (14) | 2 (22) |  |
| T factor (UICC), *n* (%) |  |  | 0.084 |  | Tumor size |  |  | 0.302 |
| T1, 2 | 11 (69) | 32 (45) |  |  | ＜50mm | 3 (43) | 7 (78) |  |
| T3, 4 | 5 (31) | 39 (55) |  |  | ≧50mm | 4 (57) | 2 (22) |  |
| Lymph node metastasis, *n* (%) |  |  | 0.472 |  | Vascular invasion |  |  | 0.615 |
| Absent | 11 (69) | 42 (59) |  |  | Absent | 4 (57) | 3 (33) |  |
| Present | 5 (31) | 29 (41) |  |  | Present | 3 (43) | 6 (67) |  |
| Lymphatic invasion (JSBPS), *n* (%) |  |  | 0.683 |  | T factor (UICC), *n* (%) |  |  | 0.596 |
| 0, 1 | 11 (69) | 45 (63) |  |  | T1, 2 | 4 (57) | 7 (78) |  |
| 2, 3 | 5 (31) | 26 (37) |  |  | T3, 4 | 3 (43) | 2 (22) |  |
| Venous invasion (JSBPS), *n* (%) |  |  | 0.046* |  | Lymph node metastasis, *n* (%) |  |  | 0.034* |
| 0, 1 | 14 (88) | 45 (63) |  |  | Absent | 7 (100) | 4 (44) |  |
| 2, 3 | 2 (12) | 26 (37) |  |  | Present | 0 | 5 (56) |  |
| Perineural invasion (JSBPS), *n* (%) |  |  | 0.634 |  | TNM stage (UICC), *n* (%) |  |  | 0.615 |
| 0, 1 | 5 (31) | 18 (25) |  |  | I, II | 4 (57) | 3 (33) |  |
| 2, 3 | 11 (69) | 53 (75) |  |  | III, IV | 3 (43) | 6 (67) |  |
| TNM stage (UICC), *n* (%) |  |  | 0.638 |  | Recurrence, n (%) |  |  | 1.000 |
| I, II | 15 (94) | 64 (90) |  |  | Absent | 4 (57) | 4 (44) |  |
| III, IV | 1 (6) | 7 (10) |  |  | Present | 3 (43) | 5 (56) |  |
| Recurrence, n (%) |  |  | 0.025* |  | UICC: Union for International Cancer Control. | | |  |
| Absent | 11 (69) | 27 (38) |  |  | * = *P* < 0.05 |  |  |  |
| Present | 5 (31) | 44 (62) |  |  |  |  |  |  |
| UICC: Union for International Cancer Control; | | |  |  |  |  |  |  |
| JSHBPS: Japanese Society of Hepato-Biliary-Pancreatic Surgery. * = P < 0.05 | | | |  |  |  |  |  |
